# Evaluation of the Antiaging Potential of the *Dendropanax morbiferus*-Derived Compound Dendropanoxide in TNF-α-Stimulated Human Dermal Fibroblasts

**DOI:** 10.3390/cimb47030188

**Published:** 2025-03-14

**Authors:** Si-Young Ahn, Sanghyun Lee, Daeyoung Kim, Sullim Lee

**Affiliations:** 1 Department of Life Science, College of Bio-Nano Technology, Gachon University, Seongnam 13120, Republic of Korea; sy990303@gachon.ac.kr; 2 Department of Plant Science and Technology, Chung-Ang University, Anseong 17546, Republic of Korea; slee@cau.ac.kr; 3 Natural Product Institute of Science and Technology, Anseong 17546, Republic of Korea

**Keywords:** *Dendropanax morbiferus*, reactive oxygen species, tumor necrosis factor-alpha, matrix metalloproteinase-1, MAPKs pathway, human dermal fibroblast

## Abstract

In this study, we investigated the antiaging potential of dendropanoxide (DP), an active compound derived from *Dendropanax morbiferus*, in human dermal fibroblasts (NHDFs) induced by Tumor Necrosis Factor-alpha (TNF-α) and in human epidermal keratinocytes (NHEKs) induced by TNF-α and interferon gamma (IFN-γ). We induced oxidative stress related to ultraviolet (UV) radiation with TNF-α and IFN-γ and then treated the cells with various concentrations of DP to evaluate its effects on reactive oxygen species (ROS) production, matrix metalloproteinase-1 (MMP-1) expression, collagen synthesis, inflammatory cytokine expression, and skin barrier protection. The results showed that DP significantly reduced ROS production, indicating its potential to alleviate oxidative stress in the skin. Additionally, DP effectively inhibited MMP-1 production, suggesting that it could prevent collagen degradation in the dermis, significantly increase the secretion of pro-collagen I, promote collagen synthesis, and protect the dermal extracellular matrix (ECM). Moreover, DP significantly reduced the expression of inflammatory cytokines IL-1β and IL-6, thereby inhibiting excessive inflammatory responses in the skin. DP also enhanced the gene expression of key factors involved in skin barrier maintenance, including *Kazal-type 5* (SPINK5), *loricrin* (LOR), *aquaporin-3* (AQP3), *filaggrin* (FLG), and *keratin 1* (KRT1), suggesting its potential to maintain and protect the skin barrier. Western blot analysis revealed that DP inhibited TNF-α-induced phosphorylation of JNK and p38, implying that DP exerts antiaging effects through the regulation of the JNK and p38 signaling pathways. Collectively, these findings suggest that DP has significant potential as an antiaging agent.

## 1. Introduction

The skin is composed of the epidermis, dermis, and subcutaneous tissue [1]. The epidermis is the outermost layer of the skin and is responsible for direct interactions with the external environment. It predominantly comprises dead keratinocytes, which generate new cells in the underlying dermis and migrate upward to the surface. The epidermis contains the stratum corneum, which serves as a barrier to protect the skin and prevent transepidermal water loss. The stratum corneum is composed of keratinocytes and plays a key role in preventing the penetration of chemicals and microorganisms while also being capable of withstanding mechanical forces [2,3,4]. The dermis lies beneath the epidermis and contains blood vessels, nerves, sweat glands, and hair follicles [5]. The dermis is rich in extracellular matrix (ECM) components, such as collagen and elastin, which provide structural integrity and elasticity to the skin and help prevent mechanical damage such as stretching or tearing [6,7,8]. The subcutaneous tissue is the deepest layer of the skin and is primarily composed of adipose and connective tissues. This is key to shock absorption, thermoregulation, and fat storage [9,10]. Of these layers, the ECM in the dermis is particularly critical to skin aging. Degradation of the fibrous proteins within the dermal ECM results in a loss of skin elasticity and the formation of wrinkles.

Skin aging is classified into intrinsic aging and extrinsic aging [11,12]. Intrinsic aging is a natural process influenced by genetic factors. As we age, the functional capacity of various bodily systems declines, and similarly, the skin undergoes progressive changes [13]. Extrinsic aging occurs when environmental factors accelerate the skin aging process [14]. Major contributing factors include ultraviolet (UV) radiation, environmental pollution, smoking, and irregular lifestyle habits [15,16]. Specifically, when the skin is exposed to UV radiation, the collagen and elastin fibers in the ECM undergo degradation, resulting in photoaging [17]. Consequently, the skin experiences a loss of elasticity, and the formation of wrinkles is accelerated.

Photoaging is primarily induced by ultraviolet A (UVA) and ultraviolet B (UVB) radiation [18]. UVA penetrates deep into the dermis, where it triggers oxidative stress and the generation of reactive oxygen species (ROS) [19]. ROS are generated when inflammatory cytokines affect the mitochondrial electron transport chain (ETC), causing oxygen molecules to combine with electrons, resulting in the production of ROS [20]. The accumulation of ROS increases oxidative damage in the skin, leading to the activation of inflammatory pathways and the production of pro-inflammatory cytokines, such as TNF-α, IL-1β, IL-6, and IL-8 [21]. UVB, which primarily affects the epidermis, causes direct DNA damage, resulting in sunburn and promoting the synthesis of inflammatory cytokines, such as TNF-α, IL-1α, IL-6, IL-8, IL-10, and IFN-γ [22,23]. These inflammatory cytokines, including IL-1α, IL-1β, and IFN, induce TNF-α expression in epidermal keratinocytes, which contributes to an inflammatory response in the skin, accelerating the aging process [24]. Inflammatory cytokines such as TNF-α and IFN-γ also affect factors that maintain the skin barrier, including Kazal-type 5 (SPINK5), loricrin (LOR), involucrin (IVL), aquaporin-3 (AQP3), filaggrin (FLG), and keratin 1 (KRT1). These proteins play crucial roles in the structural integrity and function of the skin. For example, SPINK5 helps maintain skin barrier function by inhibiting serine proteases [25,26], whereas LOR and IVL contribute to the formation of the outer layer of the skin as key components of the stratum corneum [26]. AQP3 plays an essential role in maintaining skin hydration and water homeostasis [27,28], FLG contributes to the formation of natural moisturizing factors in the skin, which is crucial for forming the skin barrier [29], and KRT1 is a key structural component that provides mechanical strength to the skin, playing an important role in the epidermis [30]. When TNF-α and IFN-γ levels increase, the expression of these important barrier proteins is inhibited, weakening the skin barrier function, increasing water loss, and making the skin more vulnerable to external irritants and pathogens. Furthermore, TNF-α upregulates the expression of matrix metalloproteinases (MMPs), enzymes responsible for the breakdown of collagen in the dermal extracellular matrix (ECM) [31]. Collagen is the most important insoluble fibrous protein found in the dermal ECM, providing strength and resilience to the skin [32]. Fibroblasts in the dermis produce pro-collagen, a precursor of collagen [33]. Although there are various types of pro-collagen, pro-collagen types I and III are particularly abundant in human skin [34,35]. Specifically, TNF-α induces the degradation of pro-collagen I, leading to loss of skin elasticity and wrinkle formation. These mechanisms of skin aging are regulated by various pathways, and the expression of MMPs is particularly regulated through the MAPKs pathway [36,37]. The MAPKs signaling pathway is activated by ROS [38,39]. The activated MAPKs pathway, in turn, activates downstream signaling pathways such as AP-1 and NF-κB, which ultimately increases the expression of MMPs [40,41]. Therefore, evaluating the activation of MAPKs serves not only to identify the mechanism of action of the sample but also as an indicator for assessing its potential antiaging properties.

Efforts to prevent skin aging have been ongoing for a long time, and among the various approaches, natural products have gained recognition for their potential as antiaging agents. Among these, we focused on the efficacy of *Dendropanax morbiferus*. *D. morbiferus* is a plant of the Araliaceae family, native to the southwestern regions of Korea. The extract of *D. morbiferus* has traditionally been used to treat dysmenorrhea, migraines, and muscle pain [42]. One promising candidate is *D. morbiferus*, a plant native to Korea that has long been used in traditional medicine for its various therapeutic properties. Recent studies have highlighted the potential of its active compound, dendropanoxide (DP), which exhibits protective effects against cisplatin-induced acute kidney injury [43]. Additionally, research findings have shown that DP alleviates thioacetamide-induced liver fibrosis and streptozotocin-induced diabetes [44,45,46]. Furthermore, DP demonstrates antioxidant and anti-inflammatory protective effects, making it an important subject of study in the context of skin aging [47]. Previous studies have demonstrated its potent anti-inflammatory effects, which are primarily mediated through the modulation of pro-inflammatory cytokine production and inhibition of the NF-κB signaling pathway [48]. For instance, DP has been shown to alleviate hepatic fibrosis and cadmium-induced hepatotoxicity by mitigating inflammatory responses and reducing oxidative stress [45,49]. Additionally, DP exhibits powerful antioxidant properties, including the scavenging of reactive oxygen species (ROS) and the enhancement of cellular antioxidant enzyme activities. These effects play a critical role in preventing oxidative damage, maintaining cell viability, and protecting tissues from degeneration. Furthermore, DP has shown neuroprotective potential by promoting neuronal survival and reducing neuroinflammation, suggesting its utility in addressing neurodegenerative conditions [50,51]. Recent investigations have also highlighted its immunomodulatory capabilities, which contribute to the regulation of immune cell function and the enhancement of immune responses, positioning it as a promising candidate for the prevention and treatment of inflammatory and immune-related diseases. Building upon these foundational studies, the current research aimed to explore the antiaging potential of DP, focusing on its effects on oxidative stress, inflammation, and extracellular matrix components in human dermal fibroblasts under photoaging-like conditions.

Therefore, in this study, we evaluated whether DP could exert antiaging effects by inhibiting ROS and protecting the ECM in a TNF-α-induced aging model using human dermal fibroblasts (NHDFs), with a particular focus on its role in maintaining the dermal ECM through collagen synthesis and organization [11]. To further investigate its effects, we assessed whether DP could prevent skin aging by inhibiting ROS and protecting the ECM in the TNF-α-induced aging model. NHDFs were treated with TNF-α to create a photoaging-like environment, followed by treatment with DP at various concentrations. To determine the antiaging potential of the compound, we measured the secretion levels of ROS, MMP-1, and collagen. Additionally, we examined its mechanism of action by Western blot analysis.

## 2. Materials and Methods

### 2.1. Chemicals

DP was isolated from *D. morbiferus*. The purity of DP was 99%, as determined by HPLC analysis from the Natural Product Institute of Science and Technology (www.nist.re.kr), Anseong, Republic of Korea.

### 2.2. Cell Culture and Treatment Preparation

Normal human dermal fibroblasts (NHDFs) and normal human epidermal keratinocytes (NHEKs) were obtained from PromoCell (Sickingenstr, Heidelberg, Germany). These cells were cultured in Dulbecco’s modified Eagle’s medium (DMEM; Corning, Manassas, VA, USA, pH 7.4) supplemented with fetal bovine serum (FBS; Atlas, Fort Collins, CO, USA) and 100 U/mL penicillin–streptomycin (PS; Gibco, Grand Island, NY, USA). The culture medium consisted of DMEM containing 10% FBS and 1% PS, and the cells were maintained in a humidified incubator at 37 °C with 5% CO_2_. For the preparation of stock solutions, the isolated DP was dissolved in dimethyl sulfoxide (DMSO; Biosesang, Seong-Nam, Republic of Korea) at a concentration of 10 mM.

### 2.3. Cell Viability

NHDFs were seeded at a density of 1 × 10^4^ cells per well in a 96-well plate and incubated for 24 h to allow for stabilization. To synchronize the cell cycle, the culture medium was replaced with serum-free DMEM, followed by an additional 24 h incubation. After medium removal, DP was administered at different concentrations, and the cells were further incubated for 24 h. Finally, a 10% Ez-Cytox solution (Dogen, Seoul, Republic of Korea) was added, and after a 1 h incubation, absorbance was measured at 450 nm using a microplate reader (SPARK 10 M; Tecan, Männedorf, Switzerland).

### 2.4. Intercellular Reactive Oxygen Species (ROS) Generation Assay

NHDFs were seeded at a density of 1 × 10^4^ cells per well in a 96-well black plate and allowed to stabilize by incubation for 24 h. To achieve cell cycle synchronization, the culture medium was replaced with serum-free DMEM, and the cells were incubated for an additional 24 h incubation. After removing the medium, DP was administered at various concentrations, and the cells were incubated for 1 h. TNF-α was then introduced at a concentration of 20 ng/mL with a subsequent incubation period of 15 min. To detect ROS production, the cells were treated with dichlorofluorescin diacetate (DCFDA; Sigma-Aldrich, St. Louis, MO, USA; CAT, no. 35845-1G) and incubated for 15 min. After staining, the cells were rinsed twice with Dulbecco’s phosphate-buffered saline (Welgene, Gyeongsangbuk, Republic of Korea) to eliminate excess DCFDA, and the fluorescence intensity was measured at excitation/emission wavelengths of 485 nm and 530 nm using a microplate reader.

### 2.5. Enzyme-Linked Immunosorbent Assay (ELISA)

NHDFs and NHEKs were seeded at a density of 2 × 10^4^ cells per well in a 48-well plate and incubated for 24 h to allow stabilization. To synchronize the cell cycle, the culture medium was replaced with serum-free DMEM; this was followed by an additional 24 h incubation. After removing the medium, DP was administered at various concentrations, and the cells were incubated for 1 h. TNF-α or TNF-α/IFN-γ was then added at a concentration of 20 ng/mL; this was followed by a 24 h incubation. The supernatant was subsequently collected for ELISA analysis. The secretion levels of MMP-1 and pro-collagen I α1 in the supernatant were quantified using the Human Total MMP-1 DuoSet ELISA kit, the Human Pro-collagen I α1 DuoSet ELISA kit, the Human IL-6 DuoSet ELISA kit, and the Human IL-1 beta/IL-1F2 DuoSet ELISA kit (R&D Systems, Minneapolis, MN, USA). The results are expressed as fold increase, and the absorbance was measured at 450 nm using a microplate reader.

### 2.6. Total RNA Isolation and Quantitative Real-Time Polymerase Chain Reaction (qRT-PCR)

NHEKs were seeded at a density of 3 × 10^5^ cells per well in a 6-well plate and incubated for 24 h to allow for stabilization. To synchronize the cell cycle, the culture medium was replaced with serum-free DMEM, followed by an additional 24 h incubation. After medium removal, DP was administered at various concentrations, and the cells were incubated for 1 h. TNF-α/IFN-γ was then added at a concentration of 20 ng/mL, followed by an additional 24 h incubation. Total RNA was then extracted from the cells using the RNeasy Mini Kit (Qiagen, Germantown, MD, USA). The extracted RNA was used for cDNA synthesis using a RevertAid First Strand cDNA Synthesis Kit (Thermo Fisher Scientific, Waltham, MA, USA). qRT-PCR was performed using the synthesized cDNA on a Quant Studio 3 Real-time PCR System (Applied Biosystems, Foster City, CA, USA) with TOPreal SYBR Green qPCR PreMIX (Enzynomics, Daejeon, Republic of Korea). The information of the primers used the experiment is shown in Table 1.

**Table 1:** Primer sequences used for real-time qPCR.

Gene	Primer Sequences	
Human SPINK5	Sense Anti-Sense	5′-GCCTGACTCTTGGAAAGAAA-3′ 3′-CAGTTGTCACTGGTTCTACA-5’
Human LOR	Sense Anti-Sense	5′-GTGGGAGCGTCAAGTACTCC-3′ 3′-AGAGTAGCCGCAGACAGAGC-5’
Human IVL	Sense Anti-Sense	5′-CAACTGGAGCTCCCAGAGCAGC-3′ 3′-AACACAGGCTGCTCCAGCTGC-5’
Human AQP3	Sense Anti-Sense	5′-TCTTTGACCAGTTCATAGGCAC-3′ 3′-GGCAGGGTTGACGGCATAG-5’
Human FLG	Sense Anti-Sense	5′-GGACTCTGAGAGGCGATCTG-3′ 3′-TGCTCCCGAGAAGATCCAT-5’
Human KRT1	Sense Anti-Sense	5′-CTGGCAGACATGGGGATAGTGTG-3′ 3′-CTGATGGTGGTGTGGCTTGTGCT-5’
Human *β-actin*	Sense Anti-Sense	5′-AGAGATGGCCACGGCTGCTT-3′ 5′-ATTTGCGGTGGACGATGGAG-3′

### 2.7. Western Blotting

NHDFs were seeded at a density of 3 × 10^5^ cells per well in a 6-well plate and incubated for 24 h to allow for stabilization. To synchronize the cell cycle, the culture medium was replaced with serum-free DMEM, followed by an additional 24 h incubation. After medium removal, DP was administered at various concentrations, and the cells were incubated for 1 h. TNF-α was then added at a concentration of 20 ng/mL; this was followed by a 15 min incubation. The cells were then harvested for the analysis of p-JNK, JNK, p-ERK, ERK, p-p38, p38, and GAPDH. For protein extraction, each well was treated with 130 μL RIPA buffer (Tech & Innovation, Gangwon, Republic of Korea) and incubated overnight at −20 °C. Lysed cells were collected and centrifuged at 13,000 rpm at 4 °C for 30 min. A 100 μL aliquot of the supernatant was collected, and the protein concentration was determined using a BCA Protein Assay Kit (Merck, Darmstadt, Germany). The Primary antibodies used in the experiment were phospho-ERK, ERK, phospho-p38, p38, phospho-JNK, JNK, and GAPDH (Cell Signaling Technology, Danvers, MA, USA) and were incubated at 4 °C for 24 h; this was followed by incubation with goat anti-rabbit IgG-HRP (Cell Signaling Technology, Danvers, MA, USA), a secondary antibody, treatment, and a 2 h incubation at room temperature. Protein bands were visualized and analyzed using immunoblotting.

### 2.8. Statistical Analysis

The data are expressed as the mean ± standard error based on at least two independent experiments. Statistical analyses were performed using a one-way analysis of variance (ANOVA) followed by Tukey’s honest significant difference test. A *p*-value of less than 0.05 was considered statistically significant.

## 3. Results

### 3.1. Chemical Structure of DP

**Figure 1:**
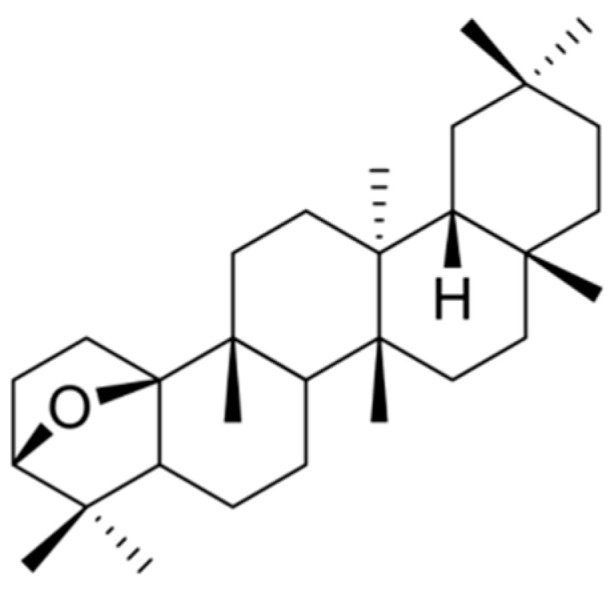
Chemical structure of dendropanoxide (DP) isolated from *Dendropanax morbiferus* (*D. morbiferus*).

DP was isolated from D. morbiferus by open column chromatography and identified by spectroscopy. The purity of DP by HPLC analysis was 99%. The structure of DP is shown in Figure 1.

### 3.2. Survival of NHDFs and NHEKs Treated with DP

**Figure 2:**
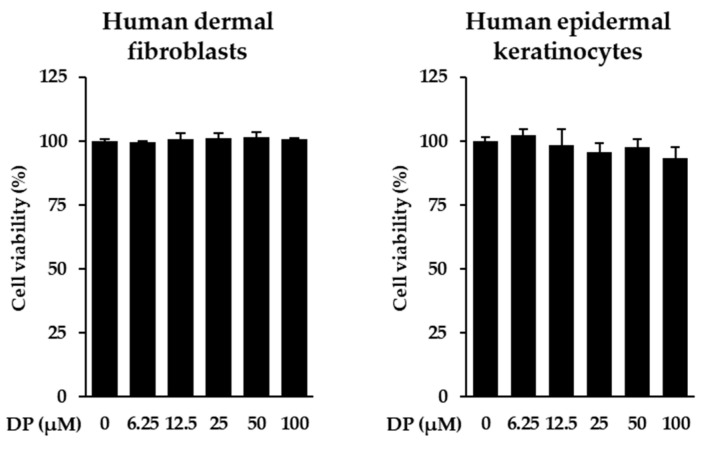
Effects of dendropanoxide (DP) on the viability of human dermal fibroblasts (NHDFs) and human epidermal keratinocytes (NHEKs). Cells were seeded in 96-well cell culture plates at a concentration of 1 × 10^4^ cells/well and cultured for 24 h. The medium was then replaced with a serum-free medium to create starvation conditions. After 24 h, the cells were treated with specific concentrations and allowed to react for 24 h. The results were obtained using the EZ-Cytox solution analysis kit. Graphs are presented as mean ± SEM.

We performed a cell viability assay to assess the toxicity of the DP to NHDFs and NHEKs at concentrations ranging from 12.5 to 100 μM. The results showed that the compound did not exhibit toxicity to the cells at these concentrations. Therefore, subsequent experiments were conducted within this concentration range (Figure 2).

### 3.3. Effects of DP on Intracellular ROS Accumulation in TNF-α-Stimulated NHDFs

**Figure 3:**
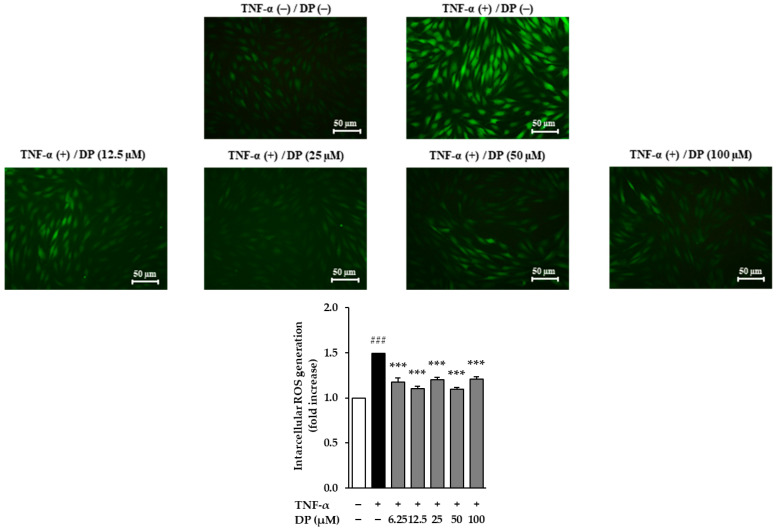
Effects of dendropanoxide (DP) on intracellular ROS accumulation in TNF-α-stimulated human dermal fibroblasts (NHDFs). We seeded HDFs at 1 × 10^4^ cells/well in 96-well black plates, incubated them for 24 h, and replaced the medium with a serum-free medium to create starvation conditions. After 24 h, the cells were treated with samples for 1 h, and the cells were exposed to 20 ng/mL TNF-α for 15 min. The cells were stained with dichlorofluorescin diacetate (DCFDA) for 15 min. The measurement of fluorescence was conducted using an EnSpire™ Multimode Plate Reader (PerkinElmer, Waltham, MA, USA). The results of intracellular ROS were presented as percentage of the vehicle control. The results were obtained through three replicate experiments, and the graphs are represented using mean ± SEM. ^###^ *p* < 0.001 vs. vehicle control. *** *p* < 0.001 vs. TNF-α-stimulated control.

TNF-α-stimulated NHDFs were used as a photoaging cell model. The increased ROS induced by TNF-α leads to elevated oxidative stress in the skin. As a result, the TNF-α-stimulated group showed a 1.49 ± 0.00-fold increase in ROS generation compared to the control group (*p* < 0.001). However, the sample-treated groups showed a significant reduction in ROS generation compared to the TNF-α-stimulated group, with ROS levels of 1.10 ± 0.03 at 12.5 μM (*p* < 0.001), 1.20 ± 0.02 at 25 μM (*p* < 0.001), 1.10 ± 0.02 at 50 μM (*p* < 0.001), and 1.21 ± 0.03 at 100 μM (*p* < 0.001) (Figure 3).

### 3.4. Effects of DP on Matrix Metalloproteinase-1 and Type 1 Pro-Collagen Secretion in TNF-α-Stimulated NHDFs

**Figure 4:**
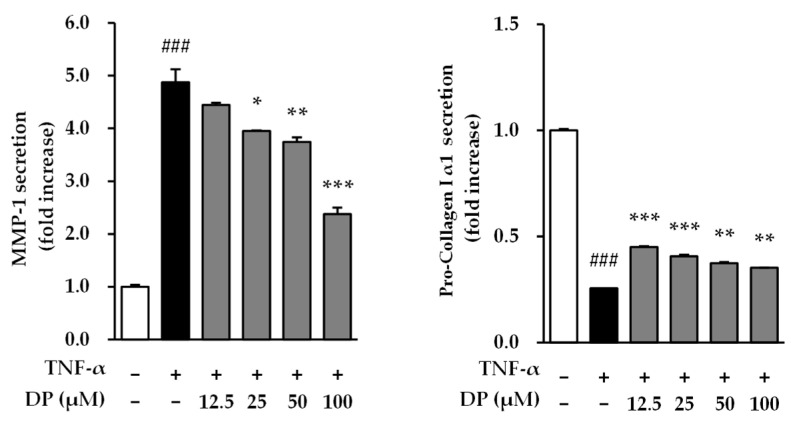
Effects of dendropanoxide (DP) on MMP-1 and pro-collagen Ι α1 secretion in human dermal fibroblasts (NHDFs). HDFs were seeded at 2 × 10^4^ cells/well in 48-well plates incubated for 24 h and replaced the medium with a serum-free medium to create starvation conditions. After 24 h, the cells were treated with samples for 1 h, and the cells were exposed to 20 ng/mL TNF-α for 24 h. MMP-1 and pro-collagen Ι α1 secretion in the cell supernatants was measured using an ELISA kit. The results of MMP-1 and pro-collagen Ι α1 secretion are presented as fold increase. The results were obtained from two replicate experiments, and the graphs are presented as the mean ± SEM. ^###^ *p* < 0.0001 vs. the vehicle control. * *p* < 0.05, ** *p* < 0.01, and *** *p* < 0.001 vs. TNF-α-stimulated control.

The inhibitory effect on the collagen-degrading enzyme MMP-1 and the secretion of collagen were evaluated in TNF-α-stimulated NHDFs. First, for MMP-1, the TNF-α-stimulated group showed a 4.88 ± 0.24-fold increase in MMP-1 secretion compared to the control group (*p* < 0.001). However, the sample-treated groups exhibited significant inhibition of MMP-1 secretion, with values of 3.95 ± 0.01 at 25 μM (*p* < 0.05), 3.74 ± 0.08 at 50 μM (*p* < 0.01), and 2.38 ± 0.12 at 100 μM (*p* < 0.001). Additionally, the effect on collagen secretion was evaluated. The TNF-α-stimulated group showed a 0.26 ± 0.00 reduction in collagen secretion compared to the unstimulated group (*p* < 0.001). However, the sample-treated groups exhibited significant increases in collagen secretion, with values of 0.45 ± 0.00 at 12.5 μM (*p* < 0.001), 0.41 ± 0.01 at 25 μM (*p* < 0.001), 0.37 ± 0.01 at 50 μM (*p* < 0.01), and 0.35 ± 0.00 at 100 μM (*p* < 0.01). Therefore, DP may have an antiaging effect by inhibiting the secretion of MMP-1 and activating collagen secretion (Figure 4).

### 3.5. Effects of DP on Inflammatory Response in TNF-α-/IFN-γ-Stimulated NHEKs

**Figure 5:**
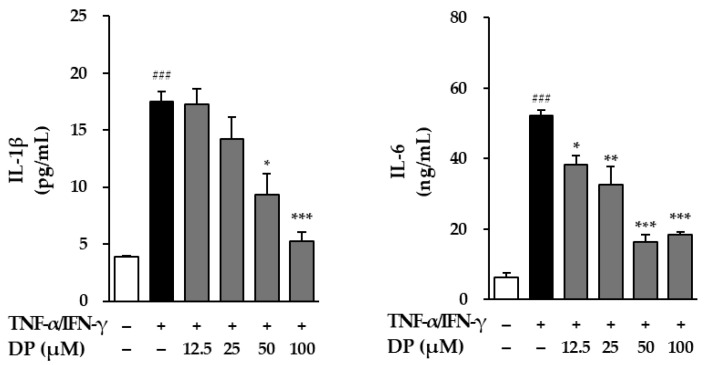
Effects of dendropanoxide (DP) oIL-1β and IL-6 secretionon in normal human epidermal keratinocytes (NHEKs). NHEKs were seeded at 2 × 10^4^ cells/well in 48-well plates and incubated for 24 h, followed by replacement of the medium with serum-free medium to create starvation conditions. After 24 h, the cells were treated with samples for 1 h and then exposed to 20 ng/mL TNF-α/IFN-γ for 24 h. IL-1β and IL-6 secretion in cell supernatants was measured using an ELISA kit. The results were obtained from two replicate experiments, and the graphs are presented as the mean ± SEM. ^###^ *p* < 0.0001 vs. the vehicle control. * *p* < 0.05, ** *p* < 0.01, and *** *p* < 0.001 vs. TNF-α-/IFN-γ-stimulated control.

The inhibitory effect on the secretion of the pro-inflammatory cytokines IL-1β and IL-6 was evaluated in TNF-α-/IFN-γ-stimulated NHEKs. First, for IL-1β, the TNF-α-/IFN-γ-stimulated group showed a 17.50 ± 0.87 in IL-1β secretion compared to the control group (3.89 ± 0.12, *p* < 0.001). However, the sample-treated groups exhibited significant inhibition of IL-1β secretion, with values of 9.35 ± 1.82 at 50 μM (*p* < 0.05) and 5.23 ± 0.85 at 100 μM (*p* < 0.001). Additionally, the effect on IL-6 secretion was evaluated. The TNF-α-/IFN-γ-stimulated group showed a 52.2 ± 1.55 in IL-1β secretion compared to the control group (6.4 ± 1.20, *p* < 0.001). However, the sample-treated groups exhibited significant reductions in IL-6 secretion, with values of 38.2 ± 2.76 at 12.5 μM (*p* < 0.05), 32.5 ± 5.24 at 25 μM (*p* < 0.01), 16.4 ± 1.98 at 50 μM (*p* < 0.001), and 18.4 ± 0.85 at 100 μM (*p* < 0.001). Therefore, DP may exert an anti-inflammatory effect by inhibiting the secretion of IL-1β and IL-6 (Figure 5).

### 3.6. Effects of DP on the Skin Barrier in TNF-α-/IFN-γ-Stimulated NHEKs

**Figure 6:**
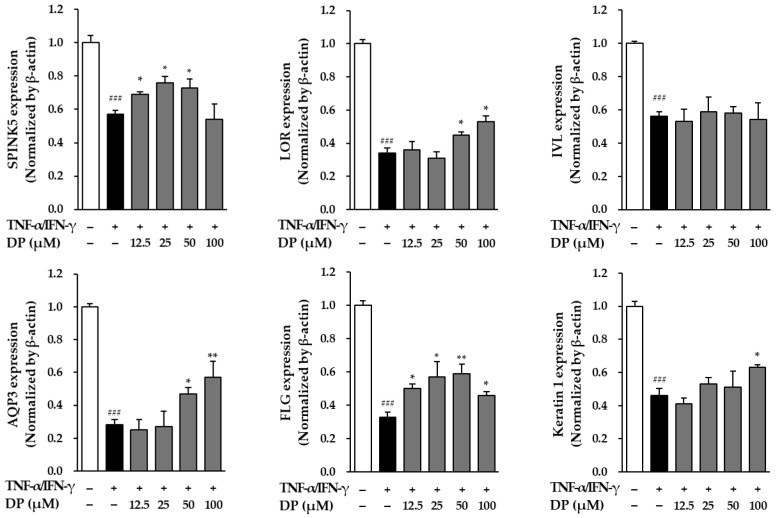
Effects of dendropanoxide (DP) skin barrier protection in normal human epidermal keratinocytes (NHEKs). NHEKs were seeded at 3 × 10⁵ cells/well in 6-well plates and incubated for 24 h to allow for stabilization. To synchronize the cell cycle, the culture medium was replaced with serum-free DMEM, and the cells were incubated for an additional 24 h. After medium removal, DP was administered at various concentrations, and the cells were incubated for 1 h. TNF-α/IFN-γ was then added at a concentration of 20 ng/mL; this was followed by an additional 24 h incubation. Total RNA was extracted from the cells and used for cDNA synthesis. qRT-PCR was performed using the synthesized cDNA to measure the gene expression of serine peptidase inhibitor *Kazal-type 5* (SPINK5), *loricrin* (LOR), *involucrin* (IVL), *aquaporin-3* (AQP3), *filaggrin* (FLG), and *keratin 1* (KRT1). The results are presented as fold increase. The results were obtained from two replicate experiments, and the graphs are presented as the mean ± SEM. ^###^ *p* < 0.0001 vs. the vehicle control. * *p* < 0.05 and ** *p* < 0.01 vs. TNF-α-/IFN-γ-stimulated control.

To investigate the effect of DP on the skin barrier in TNF-α-/IFN-γ-induced NHEKs, the gene expression of serine peptidase inhibitor *Kazal-type 5* (SPINK5), *loricrin* (LOR), *involucrin* (IVL), *aquaporin-3* (AQP3), *filaggrin* (FLG), and *keratin 1* (KRT1) was evaluated using qRT-PCR. First, for SPINK5, the TNF-α-/IFN-γ-stimulated group showed a 0.57 ± 0.02-fold decrease in SPINK5 gene expression compared to the control group (*p* < 0.001). However, the sample-treated groups exhibited a significant increase in SPINK5 gene expression, with values of 0.69 ± 0.02 at 12.5 μM (*p* < 0.05), 0.76 ± 0.04 at 25 μM (*p* < 0.05), and 0.73 ± 0.05 at 50 μM (*p* < 0.05). For LOR, the TNF-α-/IFN-γ-stimulated group showed a 0.34 ± 0.03-fold decrease in LOR gene expression compared to the control group (*p* < 0.001). However, the sample-treated groups exhibited a significant increase in LOR gene expression, with values of 0.45 ± 0.02 at 50 μM (*p* < 0.05) and 0.53 ± 0.04 at 100 μM (*p* < 0.05). For IVL, the TNF-α-/IFN-γ-stimulated group showed a 0.56 ± 0.03-fold decrease in IVL gene expression compared to the control group (*p* < 0.001), and the sample-treated groups also did not show a significant increase in gene expression. For AQP3, the TNF-α-/IFN-γ-stimulated group showed a 0.28 ± 0.03-fold decrease in AQP3 gene expression compared to the control group (*p* < 0.001). However, the sample-treated groups exhibited a significant increase in AQP3 gene expression, with values of 0.47 ± 0.04 at 50 μM (*p* < 0.05) and 0.57 ± 0.10 at 100 μM (*p* < 0.01). For FLG, the TNF-α-/IFN-γ-stimulated group showed a 0.33 ± 0.03-fold decrease in FLG gene expression compared to the control group (*p* < 0.001). However, the sample-treated groups exhibited a significant increase in FLG gene expression at all concentrations, with values of 0.50 ± 0.03 at 12.5 μM (*p* < 0.05), 0.57 ± 0.09 at 25 μM (*p* < 0.05), 0.59 ± 0.06 at 50 μM (*p* < 0.01), and 0.46 ± 0.02 at 100 μM (*p* < 0.05). Finally, for keratin 1, the TNF-α-/IFN-γ-stimulated group showed a 0.46 ± 0.05-fold decrease in keratin 1 gene expression compared to the control group (*p* < 0.001). However, the sample-treated group exhibited a significant increase in keratin 1 gene expression, with a value of 0.63 ± 0.02 at 100 μM (*p* < 0.05). This suggests that DP can effectively restore the expression of key genes related to skin barrier function in TNF-α-/IFN-γ-induced NHEKs, potentially contributing to the reduction in skin barrier disruption and cellular damage (Figure 6).

### 3.7. Phosphorylation of JNK, ERK, and p38 by DP on TNF-α-Induced NHDFs

**Figure 7:**
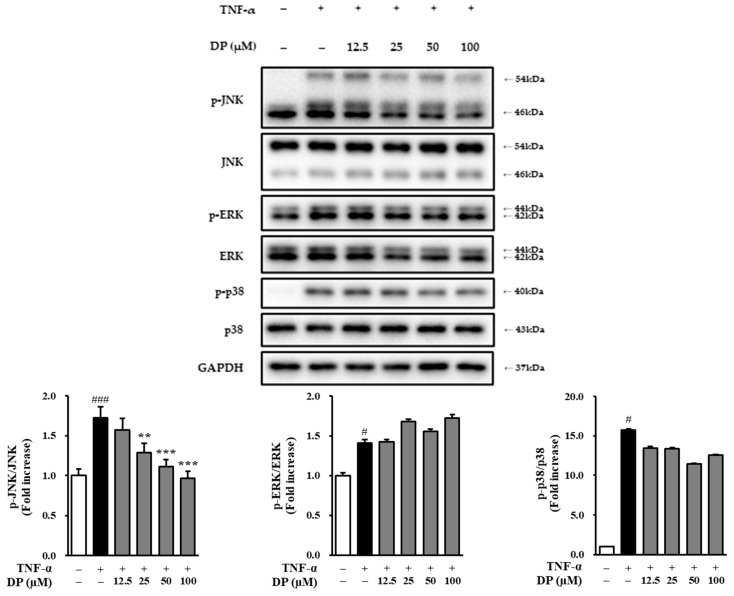
Effect of dendropanoxide (DP) on TNF-α-induced MAPK phosphorylation in normal human dermal fibroblasts (NHDFs). NHDFs were seeded at 3 × 10^5^ cells/well in 6-well plates and incubated. After 24 h, the cells were treated with 12.5, 25, 50, and 100 μM DP for 1 h and treated with 20 ng/mL TNF-α for 15 min. The cells were harvested to determine p-JNK, JNK, p-ERK, ERK, p-p38, p38, and GAPDH, treated with 120 μL of RIPA Buffer per well overnight at −20 °C. The lysed cells were collected and centrifuged at 13,000 rpm at 4 °C for 30 min. The supernatant (100 μL) was collected, and the protein was lysed using a BCA Protein Assay Kit. Primary antibodies were incubated for 24 h at 4 °C, followed by treatment with secondary antibodies for 2 h at room temperature. Protein bands were visualized and analyzed by immunoblotting analysis of p-JNK, JNK, p-ERK, ERK, p-p38, p38, and GAPDH. The results were obtained from two replicate experiments, and the graphs are presented as the mean ± SEM. ^#^ *p* < 0.05, ^###^ *p* < 0.01 vs. the vehicle control. ** *p* < 0.01 and *** *p* < 0.001 vs. TNF-α-stimulated control.

Through previous experiments, we confirmed that DP contributes to antiaging effects by modulating ROS, MMP-1, and collagen. To investigate the signaling pathways underlying these effects, we assessed the phosphorylation levels of JNK, ERK, and p38 via Western blotting. The results showed that the phosphorylation of JNK (1.73 ± 0.14-fold, *p* < 0.001), ERK (1.41 ± 0.04-fold, *p* < 0.05), and p38 (10.81 ± 0.11-fold, *p* < 0.01) was increased in the TNF-α-stimulated group compared to the control group. The sample-treated groups did not inhibit the phosphorylation of ERK compared to the TNF-α-stimulated group; however, they inhibited the phosphorylation of JNK to 1.28 ± 0.12 at 25 μM (*p* < 0.001), 1.11 ± 0.09 at 50 μM (*p* < 0.01), and 0.96 ± 0.09 at 100 μM (*p* < 0.001). Additionally, they showed a slight inhibition of p38 phosphorylation (Figure 7).

## 4. Discussion

Among the various factors that contribute to skin aging, oxidative stress induced by environmental factors, such as UV radiation, plays a pivotal role in the degradation of the extracellular matrix (ECM) of the skin, leading to loss of elasticity and the formation of wrinkles. Compounds that can reduce oxidative stress and modulate key inflammatory pathways are of great interest because of their potential to prevent or slow down skin aging. Plant-derived natural extracts and compounds are considered potential antiaging candidates because they are particularly rich in antioxidants and flavonoids. For example, *Nypa fruticans* (NF) is well known for its antioxidant properties, containing compounds such as protocatechuic acid, hydroxybenzoic acid, procyanidin B, catechin, and epicatechin. It significantly inhibits the generation of ROS, nitric oxide (NO), and prostaglandin E2 (PGE) in TNF-α- and IFN-γ-induced NHDFs, contributing to antiaging effects [52]. Additionally, one variety of *Glycine max*, Elite Line-1 (SCEL-1), has been shown to reduce the expression of MMP-1 in a 3D human skin model, preventing collagen degradation, and improve photoaging-related symptoms in a UV-exposed mouse model [53]. In addition, extracts of *Dolichos lablab* Linne (DLL) have been shown to inhibit MAPK signaling in HaCaT cells, reducing the expression of MMP-1 and MMP-9. In UVB-exposed mice, DLL extracts inhibited the activation of the MAPK/AP-1 signaling pathway, leading to a decrease in MMP expression, thereby demonstrating its potential antiaging effects [54]. Additionally, extracts from the young shoots and leaves of *Cephalotaxus harringtonia* (CH) significantly reduced ROS generation in TNF-α-induced NHDFs. The harringtonine contained in CH extracts inhibited MMP-1 and reduced collagen degradation, demonstrating its potential antiaging effects [55].

One promising candidate is *D. morbiferus*, a plant native to Korea that has long been used in traditional medicine for its various therapeutic properties. Recently, oleifolioside A, a bioactive compound isolated from *D. morbiferus*, has been shown to induce autophagy in HCT-116 human colorectal cancer cells through ERK activation [induction of autophagy by oleifolioside A in HCT-116 human colorectal cancer cells]. We focused on another compound of this plant, DP, the structure of which is shown in Figure 1. DP has the potential to exert antioxidant, anti-inflammatory, and protective effects. Previous studies have shown that DP can effectively reduce oxidative stress and inflammation in various biological models. However, its specific effects on skin aging-related factors, such as ROS generation, collagen degradation, and ECM remodeling, remain underexplored.

Therefore, we aimed to evaluate the antiaging potential of DP. Human dermal fibroblasts were stimulated with TNF-α to simulate the effects of UV-induced oxidative stress, and the cells were then treated with DP to assess ROS production, MMP-1 secretion, and collagen synthesis. Additionally, human epidermal keratinocytes were stimulated with TNF-α and IFN-γ to simulate the effects of UV-induced oxidative stress, and the cells were treated with DP to evaluate its impact on inflammation and the skin barrier. Subsequently, through Western blot analysis, we investigated the mechanism of action of DP and analyzed its effects on key signaling pathways related to the skin’s response to oxidative stress and inflammation.

As a result, DP significantly inhibited ROS induced by TNF-α at all concentrations. In TNF-α-induced NHDFs, ROS generation significantly increased by approximately 1.5-fold compared to the control group, while in the DP-treated group, it significantly decreased to a level similar to the control group (Figure 3). The reduction in ROS levels in the DP-treated group indicated that the compound may enhance the ability of the skin to manage oxidative damage, which is a key factor in the aging process. By mitigating oxidative stress, DP could contribute to preserving skin cell integrity, reducing cellular damage, and ultimately slowing down the photoaging process. Additionally, DP effectively inhibited the production of MMP-1, indicating its potential to inhibit collagen degradation in the dermis. In TNF-α-stimulated NHDFs, the secretion of MMP-1 increased by approximately 5-fold compared to the control group; however, in the DP-treated group, the secretion of MMP-1 was significantly decreased at 25, 50, and 100 μM concentrations. Additionally, in TNF-α-stimulated NHDFs, the secretion of pro-collagen I decreased by approximately 4-fold compared to the control group, while in the DP-treated group, the secretion of pro-collagen I significantly increased at all concentrations (Figure 4). Buechner, Nicole, et al. explained that MMP-1 and pro-collagen I are regulated, at least in part, by different signaling pathways [56]. The secretion of pro-collagen I is influenced by various intracellular and extracellular environmental factors in addition to MMP-1 inhibition, suggesting that, unlike the concentration-dependent inhibition of MMP-1, pro-collagen I secretion may not increase in a concentration-dependent manner. Additionally, DP significantly inhibited the production of inflammatory cytokines induced by TNF-α and IFN-γ. In TNF-α- and IFN-γ-induced NHEKs, IL-1β and IL-6 increased approximately 4.5-fold and 8-fold, respectively, compared to the control group, but in the DP-treated group, their levels were significantly decreased (Figure 5). Excessive accumulation of inflammatory cytokines such as IL-1β and IL-6 induces inflammatory responses in the skin, accelerating skin aging. Therefore, these results suggest that DP has the potential to contribute to antiaging effects by inhibiting excessive inflammatory responses in the skin. Furthermore, DP significantly increased the gene expression of SPINK5, LOR, AQP3, FLG, and keratin 1, which were reduced by TNF-α and IFN-γ (Figure 6). These results indicate that DP plays a role in inhibiting skin damage caused by external factors by increasing the expression of proteins essential for maintaining skin barrier function. Supporting this, TNF-α-induced NHDFs treated with DP showed a significant increase in the secretion of pro-collagen I. These results suggest that DP inhibits collagen degradation and promotes collagen synthesis in skin cells activated by oxidative stress and inflammatory responses induced by TNF-α. The significant decrease in the secretion of MMP-1, a collagen-degrading enzyme, indicates that DP may prevent collagen breakdown, thereby protecting the skin from structural damage. This inhibitory effect could contribute to maintaining skin elasticity and delaying wrinkle formation. Additionally, the increase in pro-collagen I secretion suggests that DP promotes skin regeneration and improves the stability of the dermal ECM (extracellular matrix). Overall, these findings provide strong evidence that DP protects skin cells in an inflammatory and oxidative stress environment, inhibiting collagen degradation and promoting collagen production, thereby exerting antiaging effects. Therefore, we concluded that DP is a compound with potential antiaging effects, and to understand its mechanism of action, we examined the phosphorylation of the MAPK pathway through Western blot analysis. The MAPK signaling pathway is activated by ROS induced by TNF-α. The activated MAPK pathway leads to the phosphorylation and activation of transcription factors AP-1 and NF-κB, which subsequently induce the production of MMP-1 and inflammatory cytokines. Therefore, inhibiting MAPK phosphorylation can contribute to the prevention of skin aging [57,58]. The Western blot results showed that DP inhibited the phosphorylation of JNK and p38 induced by TNF-α. In TNF-α-stimulated NHDFs, the phosphorylation of JNK significantly increased by approximately 1.7-fold compared to the control group, whereas a significant decrease was observed in the DP-treated group. Also, the phosphorylation of p38 significantly increased by approximately 10-fold compared to the control group, whereas a slight decrease was observed in the DP-treated group (Figure 5). In TNF-α-stimulated conditions, where the phosphorylation of both p38 and JNK significantly increased, DP effectively reduced these increases, suggesting its ability to regulate stress response mechanisms and inflammatory pathways involved in skin aging. Specifically, DP showed a stronger inhibitory effect on JNK phosphorylation, reducing it in a concentration-dependent manner, which underscores its role in mitigating cellular stress responses more effectively through this pathway. By inhibiting the phosphorylation of JNK, DP may help prevent the degradation of structural proteins and the activation of pro-inflammatory mediators [59,60]. Similarly, the reduction in p38 phosphorylation highlights its role in modulating inflammatory responses and maintaining cellular homeostasis [61,62]. We propose that the selective inhibition of JNK and p38 phosphorylation by DP may be associated with MEK4 (MKK4), a key regulator of JNK activation. Previous studies have demonstrated that MEKK4 not only activates MKK4 and MKK7 but also stimulates MKK3 and MKK6, thereby initiating the p38 MAPK signaling pathway. Furthermore, it is well established that ROS-mediated oxidation of thioredoxin leads to its dissociation from ASK-1, which in turn activates ASK-1 and subsequently triggers the JNK and p38 signaling cascades. Based on these findings, we hypothesize that DP modulates these mechanisms, collectively contributing to its selective inhibition of JNK and p38 phosphorylation. However, this remains a hypothesis, and further experimental investigations are required to elucidate the precise molecular mechanisms underlying these effects [38].

**Figure 8:**
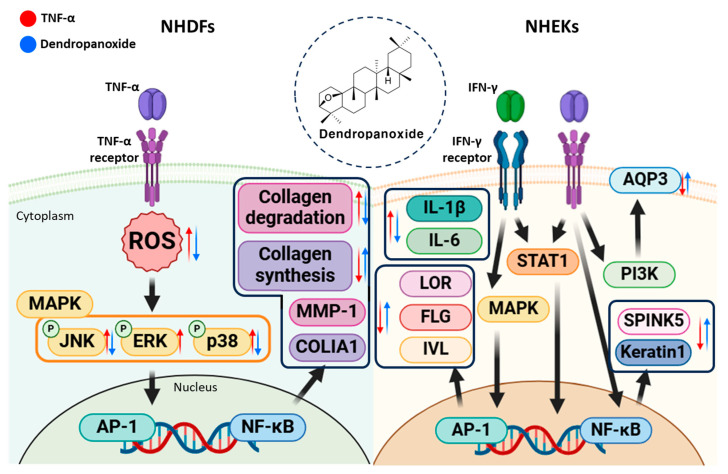
Schematic illustration of the potential protective effect of dendropanoxide (DP) in TNF-α-induced normal human dermal fibroblasts (NHDFs).

Therefore, these findings support the hypothesis that DP may alleviate photoaging and other aging-related processes by simultaneously modulating multiple signaling pathways, such as JNK and p38. These dual inhibitory effects significantly contribute to their potential antiaging properties. The experimental results were synthesized and are presented in a schematic illustration to highlight the antiaging mechanisms of DP (Figure 8).

## 5. Conclusions

In conclusion, this study confirmed that DP, a compound extracted from *D. morbiferus*, has antiaging effects. DP significantly inhibits ROS production induced by TNF-α, reduces the secretion of MMP-1, and promotes pro-collagen I secretion, which plays a crucial role in maintaining the dermal ECM. Specifically, DP suppressed ROS generation in an oxidative stress environment caused by TNF-α treatment, inhibited the production of MMP-1, an enzyme involved in collagen degradation, and promoted pro-collagen I secretion, which contributed to maintaining skin elasticity and preventing collagen degradation. In addition, DP significantly reduced the production of inflammatory cytokines (IL-1β and IL-6) induced by TNF-α and IFN-γ, suggesting that it may alleviate excessive inflammatory responses in the skin. DP also increased the expression of SPINK5, LOR, AQP3, FLG, and keratin 1, which are essential for maintaining skin barrier function. Western blot analysis showed that DP inhibited the phosphorylation of JNK and p38, key components of the MAPK signaling pathway, which are involved in stress and inflammatory responses related to skin aging. These findings indicate that DP may reduce photoaging and other aging-related processes by regulating multiple signaling pathways such as JNK and p38, contributing significantly to its antiaging properties. However, this study was limited to experiments at the cellular level, and further research is needed to validate the effects of DP through in vivo studies. Moreover, clinical studies are required to assess the impact of DP at various concentrations on actual human skin.

## Data Availability

This published article contains all the data created or analyzed during this study. Any additional data or information can be made available by the corresponding author upon request.

## Abbreviations

The following abbreviations are used in this manuscript:

AP-1	Activator Protein 1
AQP3	Aquaporin 3
*D. morbiferus*	*Dendropanax morbiferus*
*DP*	Dendropanoxide
ECM	Extracellular Matrix
FLG	Filaggrin
IFN-γ	Interferon-Gamma
IL-1β	Interleukin-1β
IL-6	Interleukin-6
IL-8	Interleukin-8
IVL	Involucrin
JNK	c-Jun N-Terminal Kinase
LOR	Loricrin
MAPKs	Mitogen-Activated Protein Kinases
MMP-1	Matrix Metalloproteinase-1
NF-κB	Nuclear Factor Kappa-Light-Chain-Enhancer of Activated B Cells
NHDFs	Normal Human Dermal Fibroblasts
ROS	Reactive Oxygen Species
SPINK5	Serine Peptidase Inhibitor, Kazal-Type 5
TNF-α	Tumor Necrosis Factor-Alpha
UV	Ultraviolet
UVA	Ultraviolet A
UVB	Ultraviolet B
